# Pharma TARP: A Troubled Asset Relief Program for Novel, Abandoned Projects in the Pharmaceutical Industry

**DOI:** 10.1100/tsw.2011.61

**Published:** 2011-02-14

**Authors:** Tamas Bartfai, Graham Vaughan Lees

**Affiliations:** ^1^ Molecular and Integrative Neurosciences Department, The Scripps Research Institute, La Jolla, CA, USA; ^2^ TheScientificWorld, Kirkkonummi, Finland

**Keywords:** TARP, pharmaceutical industry, society, Pfizer, Merck, GlaxoSmithKline, GSK, NIH, NICE, Wall Street, FDA, EMA, government, orphan drugs, intractable epilepsy, cancer pain, neuropathic pain, obesity, type-2 diabetes, schizophrenia

## Abstract

Within days of each other, Pfizer, Merck, and GlaxoSmithKline announced that they will focus on a few therapeutic areas only and abandon others entirely. Pfizer alone will close well over a hundred drug development projects that have reached two-thirds of the way to launch. The programs are deemed to be too risky and not lucrative enough for Big Pharma in the current climate. Society has a real need for the drugs that are no longer going to be developed for, among others, drug-resistant epilepsy, neuropathic and cancer pain, type-2 diabetes, obesity, and schizophrenia. The authors propose a radical response by the U.S. government and the National Institutes of Health to rescue these abandoned projects, and to continue selected programs for drug approval by the U.S. Food and Drug Administration and the European Medicines Agency. The investment required is small compared to the Troubled Asset Relief Program bank bail out, but the return on investment in financial terms and in satisfying societal needs makes this proposal attractive.

Within days of each other, Pfizer, Merck, and GlaxoSmithKline (GSK) announced that they will focus on a few therapeutic areas only and abandon others entirely[1,2,3,4]. Pfizer alone will close well over a hundred drug development projects that have reached two-thirds of the way to launch, i.e., have shown safety and some clinical efficacy in phase 1 and phase 2 trials. The medical needs and the scientific bases have not changed, but these advanced projects are no longer considered commercially lucrative. The companies' financial horizons have changed, and present management is not interested or feels that it cannot work on projects that will make little difference to the income, profit, and stock price.

It has become clear that clinical trials will be much longer and more expensive than originally expected when these projects started in 2005–2007. This new rationale of either rapid and large return on investment or no continuation of the drug development program applies equally to all kinds of drugs that our society would need. For example, it leads to the closure of projects that aimed to create new drugs for treatment-resistant epilepsy as these would "only" generate $300 million/year. Drugs that may prevent or slow down the progression of Alzheimer's disease (AD) are also abandoned even though they would be expected to be multibillion-dollars-per-year drugs. This is because we now diagnose the disease earlier and the efficacy of a putative preventive drug in AD may now take at least 3–5 years instead of 12–18 months to evaluate in clinical trials[5]. Drugs badly needed to treat neuropathic and cancer pain[6] are dropped because the failure rate of putative new pain drugs has been over 90% in the past 10 years[7,8].

Thus, these programs are projected to be overly risky and would each cost $400–800 million dollars to evaluate in phase 3 trials[9]. So these Big Pharma managements are closing these projects and have announced stock buy-back for billions of dollars at Pfizer, Merck, and GSK, lest, perhaps, they lose their own jobs, much like the Pfizer CEO a month ago[10]. They will buy back the stock as a sure way of increasing the stock price. It is like insiders buying the stock in order to advertise confidence so that Wall Street reacts favorably. They will let go of the experts, assembled over decades, to work on these therapeutic areas[11]. Cutting jobs has a history of being welcomed by Wall Street[12,13].

However, society badly needs some of the new medicines that are now the casualties of these project closures for insufficient financial gain in the new economic climate. The medical and scientific rationales have not changed; they remain the same as when the projects were started. Because the medical needs persist, we, as a society, should pick up those phase 1 and 2 projects that promise a therapy in a disease where none exists today or where the novel therapy may be an improvement over existing therapy. Society should not rescue all of them, however. Those that were aimed at producing "me-too" medicines with essentially the same therapeutic benefits as some already-approved drugs might be safely abandoned.

It is estimated that of the several hundred projects closed, there are at least 30–50 that should result in at least five major drugs within 5 years to treat diseases where we currently do not have any or we have only inferior drugs. We should run the clinical trials in some government-directed institutions (e.g., the National Institutes' of Health Translational Medicine Branches[14]) using "TARP-like" funds, i.e., a *de novo* Troubled Asset Relief Program, for these phamaceutical programs. The Women's Health Initiative in the U.S.[15,16,17] and the National Institute for Health and Clinical Excellence (NICE)[18] in the U.K. show that we can obtain superbly important and very high-quality information from government-funded large and long clinical trials. The results would still be judged by the U.S. Food and Drug Administration (FDA) and the European Medicines Agency (EMA) just as for any private company trials. The cost of conducting the 30 trials will be approximately $15 billion over 5 years, yet the income from the five, say, resulting approved drugs, is estimated to range between $300 million and $2 billion/year *each*. Thus, the revenues over only about 3 years will pay for both the successful and failed trials. The approved drugs should be produced by the company that owns the intellectual property (IP) or, if they are not interested, then by some of the many generic drug companies. Of course, it may be that if the IP rights were made available at no cost, some smaller pharmaceutical companies might be able to take up the cause and develop drugs to commercialization. However, it is the authors' view that government intervention at an early stage is essential in order to avoid opportunities being lost and commercial interests alone determining the outcome of these now-abandoned projects aimed at novel therapies in key diseases with presently less favorable financial prospects. Only the government would have the power to open discussions with Big Pharma and it will not only be the three companies we have specifically mentioned herein that will be abandoning drug development programs. The first step is to intervene and set up an expert think tank to determine the process, and then to evaluate the dropped projects. Government would not only recoup its financial expenditure over the years, but would also improve public health, which is one of its prime responsibilities. We have not estimated the medical cost savings that would accrue from being able to treat diseases more effectively, although they would be considerable. We have not discussed how the orphan drug program or the pediatric extension program type status for these drugs could be used to assist commercial success because these are details of the negotiations. We wish primarily to call attention to the damaging delay or complete loss of promising new drugs in areas of medical need.

These Pharma giants and their British counterpart GSK have announced that the R&D cuts of billions of dollars will be used to buy back stock[3], showing that these are not companies at the brink of collapse. However, the companies are also not obligated to spend their funds on projects that they no longer believe will improve their stock value in the short run. In this proposed form of public-private partnership, we should use government funds to make sure that, for society, important areas of drug development do not come to a halt. We understand this when it comes to vaccine production, which is so important for public health that governments have to provide a guaranteed market for Big Pharma and the promise to buy hundreds of millions of doses.

When the highly profitable enzyme replacement therapy, which was a virtual monopoly of Genzyme until a year ago, came into production problems, the patients who were dependent for their life on the product had none, and the government had to accelerate approval of competing products and to switch to suppliers of alternative therapies[19,20,21].

There are other aspects of this situation worth mentioning. Pharma is also one of the most high-tech industries with product development times of 10–12 years and with the highest per capita employment of PhD-level scientists, where product candidates and products are fiercely protected by patents. When these companies stop developing these putative drugs in disease areas such as pain, AD, allergy, virology, etc., then some of their well-protected research results block the way for others; their well-written and defended patents stand in the way of others developing a promising therapy.

We have to recognize that one crucial aspect of the problem is that Pharma is not just any industry. It is an ethical industry, or we would like it to be an ethical industry, which, while the investors profit from innovative products, does not forget that it fulfills society’s aspiration to diminish human suffering. If it does not fulfill society’s needs, it will not be successful. Because of its critical role in society, it is very much the government’s business to step in when it is unable or fails to fulfill society’s needs.

In 2011, the Obama administration recognized that AD, which affects one of two people over 80 years of age, will present a major health, economic, and societal problem or crisis. It must act as governments have done earlier with the “war on cancer” or the campaign against HIV, and must spend on basic research such as that which provides drug companies with drug targets. Basic research is almost nonexistent anymore in Big Pharma and the government, or governments, must find means of financially and politically encouraging the development of drugs in major diseases like AD, pain therapies, obesity, type-2 diabetes, and schizophrenia. The example of HIV, where social and political pressure and government and industry research turned a deadly disease into a chronic disease, and the encouraging example of increasing progress in treating different forms of cancer, show that government spending on basic research and political will can make private companies produce remarkable medicines for the benefit of all. The substantial NIH budget of $29.5 billion in 2009 is to be compared to the industry R&D of $92 billion in 2009[22]. Taking on the promising, but stopped, projects from Pharma would be another important step, as important as helping out the banks was. It would also be less expensive and give a more sure return on tax-payer investment.
